# Deep learning-enhanced zero echo time silent brain magnetic resonance imaging in infants without sedation

**DOI:** 10.1007/s00247-025-06413-0

**Published:** 2025-11-12

**Authors:** Chanyoung Rhee, Jae-Yeon Hwang, Jae Won Choi, Yeon Jin Cho, Seunghyun Lee, Jung-Eun Cheon, Young Hun Choi

**Affiliations:** 1 Department of Radiology, Seoul National University Hospital, Seoul, Republic of Korea; 2 Department of Radiology, Seoul National University College of Medicine, 101 Daehak-ro, Seoul, 03080 Republic of Korea

**Keywords:** Deep learning, Feed-and-wrap technique, Magnetic resonance imaging, Pediatric, Sedation, Zero echo time

## Abstract

**Background:**

Reducing acoustic noise is essential in infant brain magnetic resonance imaging (MRI) to minimize the need for sedation. Deep learning (DL)–based MRI reconstruction may enhance the image quality of the zero echo time (ZTE) silent sequence.

**Objective:**

To evaluate the effect of DL-based reconstruction on the image quality of ZTE infant brain MRI using the feed-and-wrap technique, compared to conventional MRI with sedation.

**Materials and methods:**

This retrospective study included 78 infants (postmenstrual age ≤16 months) who underwent brain MRI between January 2022 and December 2024. The control group underwent sedated 3-dimensional T1-weighted magnetization-prepared rapid gradient-echo (MPRAGE) imaging. The experimental group underwent unsedated inversion recovery-prepared ZTE imaging with and without DL-based reconstruction (ZTE-DL and ZTE, respectively), using the feed-and-wrap technique. Three radiologists independently rated five image quality metrics using a 5-point Likert scale. Signal uniformity was assessed by the coefficient of variation across eight brain regions. Differences among sequences were analyzed using the Mann–Whitney *U* test with Bonferroni correction. Interrater agreement was assessed using Cohen’s kappa coefficient.

**Results:**

ZTE-DL had the highest scores for noisiness, gray-white matter differentiation, artifacts, and overall image quality. ZTE-DL showed no significant differences from MPRAGE except for reduced noisiness, while significantly outperforming ZTE across all metrics (all *P*<0.017). Lesion conspicuity did not differ significantly among the groups. Interrater agreement was substantial (*κ*>0.6) for most metrics. Signal uniformity was greatest in ZTE-DL for gray and white matter (all *P*<0.001); no difference was observed between ZTE-DL and ZTE for cerebrospinal fluid (*P*=0.721).

**Conclusion:**

DL-based MRI reconstruction improved ZTE image quality and provided comparable image quality to MPRAGE, potentially reducing the need for sedation in infant brain MRI.

**Graphical abstract:**

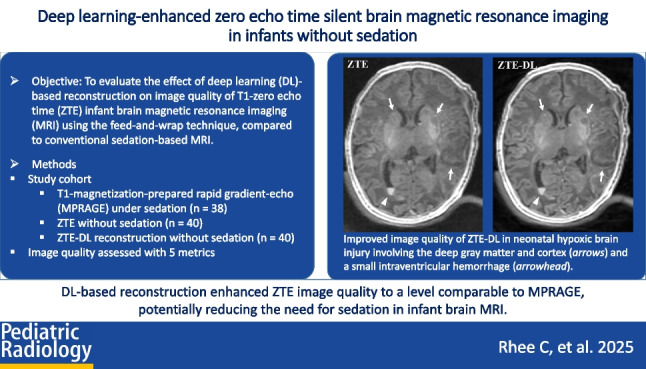

## Introduction

Magnetic resonance imaging (MRI) is a nonionizing technique that provides high-resolution images of anatomical structures and physiological features of the brain in pediatric patients [1]. In neonates and infants, early detection of congenital malformations or prematurity-related injuries using MRI is essential for predicting neurodevelopmental outcomes [2]. Achieving high-quality brain MRI requires minimizing patient motion, which often necessitates sedation or anesthesia in infants. However, these interventions may have neurotoxic effects on the developing brain, prompting efforts to reduce scan times through techniques such as single-shot fast spin echo, radial k-space sampling, and compressed sensing [3–5].

Silent MRI has recently emerged as a technique to address these challenges by reducing acoustic noise while maintaining diagnostic performance comparable to conventional MRI in evaluating pediatric brain myelination [6–8]. The zero echo time (ZTE) sequence is fundamental for silent MRI. It employs radial center-out encoding to run gradients continuously without switching, capturing data immediately after radiofrequency excitation (echo time (TE) ≈ 0), thereby preserving short T2 signals [9]. Nevertheless, ZTE uses a short repetition time (TR) for rapid data acquisition and a low flip angle, which inherently reduces the signal-to-noise ratio (SNR) and limits contrast in soft tissues with long T2 relaxation times. As a result, image quality is often lower compared to conventional MRI. Additionally, the radial acquisition method complicates data processing and image reconstruction compared to Cartesian techniques [10, 11].

Recent advances in deep learning (DL)-based MRI reconstruction have improved image quality by enhancing SNR and reducing artifacts, while also accelerating reconstruction through k-space learning, image-domain learning, or direct mapping [12–15]. Several studies have demonstrated the clinical utility of DL-based denoising, including applications in pediatric neuroimaging [16–19]. These advances have enabled various MRI vendors to apply DL to k-space data to generate high-quality images [20, 21]. Consequently, applying DL to ZTE imaging of the infant brain may enhance image quality while reducing acoustic noise and scan time, potentially reducing the need for sedation.

This study aimed to assess the effect of DL-based reconstruction on the image quality of T1-ZTE infant brain MRI acquired using the feed-and-wrap technique, in comparison with conventional MRI performed under sedation.

## Materials and methods

This study received approval from our Institutional Review Board (*Seoul National University Hospital *IRB No. H-2505-045-1637), and the need for patient informed consent was waived.

### Study cohort

This retrospective study included 38 consecutive patients (21 boys; gestational age at birth, 29.6±5.1 weeks; postmenstrual age at imaging, 39.4±3.3 weeks) who underwent brain MRI between January 1 and July 31, 2022, as the control group. Chloral hydrate was administered orally at a dose of 50 mg/kg for sedation. The experimental groups consisted of 40 consecutive patients (15 boys; gestational age at birth, 28.8±4.4 weeks; postmenstrual age at imaging, 39.9±5.5 weeks) who underwent brain MRI using the feed-and-wrap technique without sedation between April 1, 2024, and December 31, 2024 [22]. Inclusion criteria were infants with a postmenstrual age of 16 months or younger who underwent brain MRI, including a 3-dimensional (3D) T1-weighted sequence and were admitted to the neonatal intensive care unit (NICU). Demographics of the control and experimental groups are summarized in Table 1.

**Table 1:** Demographics of the study cohort

Characteristic	MPRAGE	ZTE/ZTE-DL	*P*-value
Number of patients	38	40	-
Sex (male:female)	21:17	15:25	0.178
Gestational age at birth (weeks)	29.6±5.1	28.8±4.4	0.406
Postmenstrual age at imaging (weeks)	39.4±3.3	39.9±5.5	0.734
**MRI findings**			
Intracranial hemorrhage	6	8	0.770
Hypoxic brain injury	2	3	>0.99
Congenital brain malformation	2	3	>0.99
Congenital metabolic disease	1	0	0.487
Cephalohematoma	1	0	0.487
Unremarkable	26	25	0.639

Data are presented as the mean±standard deviation or as the number of participants

* MPRAGE* magnetization-prepared rapid gradient-echo, *MRI* magnetic resonance imaging, *ZTE* zero echo time, *ZTE-DL* zero echo time with deep learning-based reconstruction

### Magnetic resonance imaging protocol

All MRI was conducted on a 3-T scanner (SIGNA Premier; GE Healthcare, Waukesha, WI) equipped with a 48-channel head coil. The brain MRI sequences for the control group included 3-D T1-weighted magnetization-prepared rapid gradient-echo (MPRAGE), T2-weighted fast spin echo, susceptibility-weighted imaging, axial diffusion-weighted imaging (DWI), and apparent diffusion coefficient (ADC) maps. The experimental groups underwent 3-D T1-weighted inversion recovery-prepared ZTE imaging with and without DL reconstruction, along with T2-weighted fast spin echo, axial DWI, and ADC maps.

For the control group, MPRAGE images were processed using intensity filter A (ClariView; GE Healthcare), which applies mild sharpening and denoising. In the experimental groups, inversion recovery-prepared ZTE images were processed with intensity filter B (ClariView; GE Healthcare), which applies stronger sharpening and denoising. The ZTE k-space data were reconstructed using a vendor-supplied prototype DL-based reconstruction algorithm (AIR™ Recon DL; GE Healthcare), referred to as ZTE-DL [20]. The MRI parameters for MPRAGE, ZTE, and ZTE-DL are presented in Table 2.

**Table 2:** Magnetic resonance imaging acquisition parameters

Parameter	MPRAGE	ZTE/ZTE-DL
Repetition time (ms)	2,350–2,450	1,098
Echo time (ms)	2.4	0.0
Flip angle (°)	15.0	5.0
Preparation time (ms)	1,200	450
Recovery time (ms)	900	200
Bandwidth (kHz)	±31.25	±25.00
Spokes per segment	-	512
Field of view (mm)	180×180	180×180
Slice thickness (mm)	1.0	1.0
Slice spacing (mm)	1.0	1.0
Matrix size	180×180	180×180
Number of acquisitions	1	3
Post-processing filter	Intensity filter A	Intensity filter B
Deep learning reconstruction	No	No (ZTE)/yes (ZTE-DL)

*MPRAGE* magnetization-prepared rapid gradient-echo, *ZTE* zero echo time, *ZTE-DL* zero echo time with deep learning-based reconstruction

### Deep learning reconstruction algorithm

The DL-based reconstruction algorithm processed raw k-space data to reduce noise and truncation artifacts while enhancing edge sharpness, thereby improving image quality over conventional methods [20]. The convolutional neural network comprised approximately 4.4 million trainable parameters across 10,000 kernels, used ReLU activations without bias terms, and was designed for scale-invariant and effective blind denoising across varying noise levels. Supervised training was performed using paired datasets consisting of near-perfect high-resolution images and synthetically degraded conventional images. Data augmentation, including geometric and intensity-based transformations with added noise, yielded a dataset of four million unique samples. Training was completed in a single epoch comprising four million iterations using the ADAM optimizer [23]. Although originally developed for 2-D data, the algorithm was recently extended to 3-D to reduce noise and ringing artifacts in all spatial directions [24–26].

### Qualitative analysis

Three radiologists (*BLINDED *with 2 years, 14 years, and 15 years of experience in pediatric brain MRI interpretation) independently evaluated three imaging sequences (MPRAGE, ZTE, and ZTE-DL) using a 5-point Likert scale. The five image quality metrics were image noisiness, gray-white matter differentiation, artifacts, lesion conspicuity, and overall image quality. The evaluations were performed using the INFINITT PACS 7.0 system (INFINITT Healthcare Co., Ltd., Seoul, South Korea). Reviewers were not restricted in window level settings, evaluation time, or image scrolling. A radiologist (*BLINDED*), who did not participate in the image quality assessment, randomly arranged images from the three sequences. Reviewers received these images in random order and were blinded to patient information and scan parameters that could reveal the imaging sequence. They were also blinded to each other’s assessments.

Image noisiness, gray-white matter differentiation, and overall image quality were rated on the following scale: 1, unacceptable; 2, poor; 3, acceptable; 4, good; and 5, excellent or ideal. Artifacts, including truncation and motion artifacts, were scored as 1, unreadable artifact, non-diagnostic images; 2, severe artifact, degraded but interpretable; 3, moderate artifact, some effect on diagnostic quality but not severe; 4, minimal artifact, no significant effect on diagnostic quality; and 5, no visible artifact. For patients with pre-annotated lesions (MPRAGE, 12/38 [31.6%]; ZTE and ZTE-DL, 14/40 [35.0%]), lesion conspicuity was classified as follows: 1, not visible; 2, blurred but detectable; 3, moderately clear; 4, well visualized; and 5, highly distinct. Reviewers were provided with lesion locations.

### Quantitative analysis

Signal uniformity in white matter, gray matter, and cerebrospinal fluid (CSF) regions was assessed using the coefficient of variation for quantitative analysis. One pediatric radiologist (*BLINDED*) placed eight regions of interest (ROIs) on axial images from all three sequences: two in the white matter (bilateral centrum semiovale), four in the deep gray matter (bilateral putamen and thalami), and two in the CSF (bilateral body of the lateral ventricle). The axial plane was carefully chosen to best display the target structures while avoiding non-parenchymal areas such as blood vessels, sulci, and cisterns. Each ROI had an area of at least 5 mm^2^. For each region, the coefficient of variation was calculated as the standard deviation divided by the mean signal intensity and then averaged for each tissue type. These tissue-specific values were compared across the three sequences.

### Statistical analysis

Demographic comparisons between groups were performed using the Mann–Whitney *U* test, except for sex, which was analyzed with the chi-squared test. Fisher’s exact test was used to compare the proportions of MRI findings between groups. A *P*-value <0.05 was considered statistically significant. For qualitative and quantitative comparisons of the three sequences, the Mann–Whitney *U* test with Bonferroni correction was applied, with a significance threshold set at *P*<0.017. Interrater agreement among the three readers was assessed using pairwise weighted Cohen’s kappa tests. Agreement levels were interpreted as follows: 0.20 or below, poor; 0.21–0.40, fair; 0.41–0.60, moderate; 0.61–0.80, substantial; and 0.81–1.00, almost perfect. All statistical analyses were conducted using Python (v.3.13.2; Python Software Foundation, Wilmington, DE) along with the pandas (v.1.1.5), SciPy (v.1.6.0), and scikit-learn (v.1.1.3) libraries.

## Results

### Qualitative analysis

**Fig. 1:**
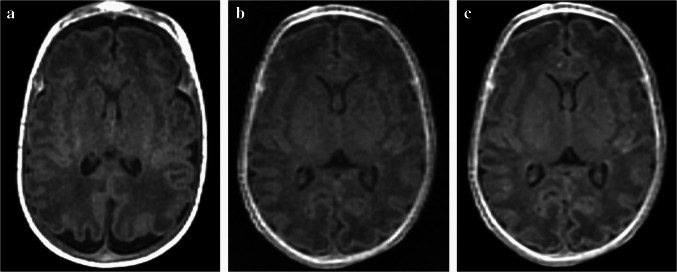
Representative brain magnetic resonance images from magnetization-prepared rapid gradient-echo (MPRAGE), zero echo time (ZTE), and zero echo time with deep learning-based reconstruction (ZTE-DL) sequences. **a** Axial T1-weighted MPRAGE image of an 8-week-old girl (born at 27+5 weeks of gestation and imaged at 36 weeks postmenstrual age). **b**, **c** Axial T1-weighted ZTE images without (**b**) and with (**c**) deep learning reconstruction of a 7-week-old girl (born at 28 weeks of gestation and imaged at 35 weeks postmenstrual age). The median overall image quality score was 4 in MPRAGE (**a**) and ZTE-DL (**c**), and 3 in ZTE (**b**). *MPRAGE* magnetization-prepared rapid gradient-echo, *ZTE* zero echo time, *ZTE-DL* zero echo time with deep learning-based reconstruction

**Fig. 2:**
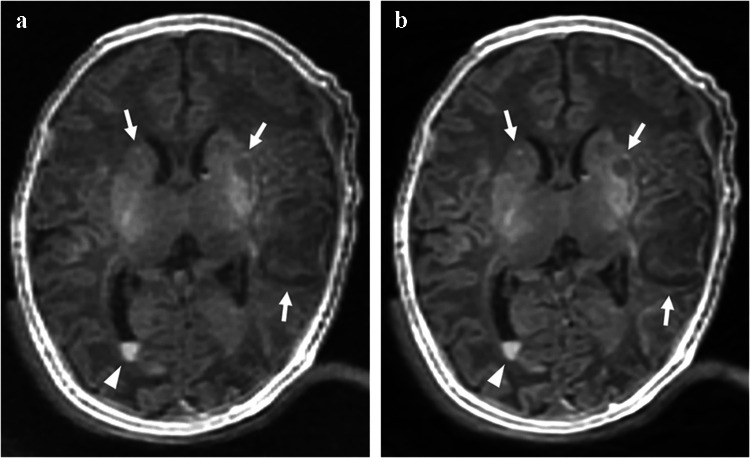
Axial T1-weighted images of a 2-week-old girl (born at 39+1 weeks of gestation and imaged at 41 weeks postmenstrual age) with hypoxic-ischemic encephalopathy*. ***a** Zero echo time (ZTE). **b** Zero echo time with deep learning-based reconstruction (ZTE-DL). Images show hypoxic brain injury involving the deep gray matter and cortex (*arrows*). A small intraventricular hemorrhage is also present (*arrowhead*). ZTE-DL (**b**) shows improved overall image quality, decreased noise and artifacts, and better lesion conspicuity compared to ZTE (**a**). *ZTE* zero echo time, *ZTE-DL* zero echo time with deep learning-based reconstruction

**Fig. 3:**
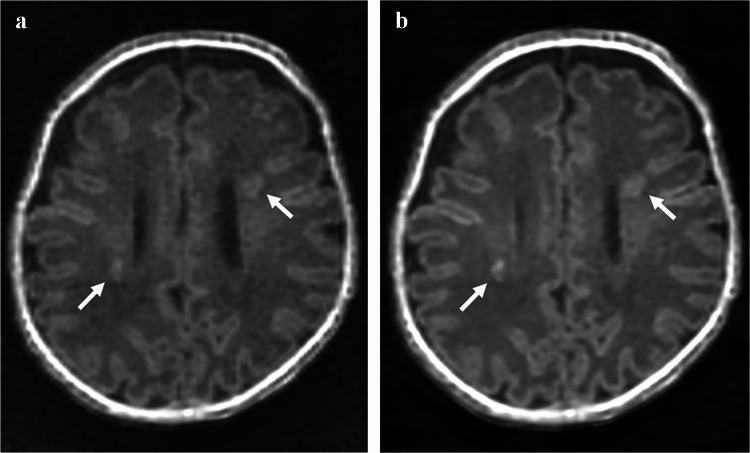
Axial T1-weighted images of a 14-week-old boy (born at 25 weeks of gestation and imaged at 39 weeks postmenstrual age) with white matter brain injury.** a** Zero echo time (ZTE). **b** Zero echo time with deep learning-based reconstruction (ZTE-DL). Images show multifocal T1-hyperintense lesions in the bilateral periventricular white matter (*arrows*). ZTE-DL (**b**) shows improved overall image quality, reduced noise, enhanced gray-white matter differentiation, and better lesion conspicuity, compared to ZTE (**a**). *ZTE* zero echo time, *ZTE-DL* zero echo time with deep learning-based reconstruction

The mean and standard deviation of scores for the five image quality metrics for each sequence are summarized in Table 3, with representative axial images shown in Fig. 1. For image noisiness, gray-white matter differentiation, artifacts, and overall image quality, ZTE-DL achieved the highest mean scores across all three readers, followed by MPRAGE and ZTE. When comparing MPRAGE and ZTE, all readers reported statistically significant differences in gray-white matter differentiation and overall image quality, while only reader 3 noted significant differences in noisiness and artifacts. No significant differences were observed between MPRAGE and ZTE-DL across all readers (all *P*>0.017), except for noisiness, which was rated significantly different by readers 1 and 2. Compared to ZTE, ZTE-DL showed significantly higher mean scores for image noisiness, gray-white matter differentiation, artifacts, and overall image quality across all readers (all *P*<0.017) (Figs. 2 and 3). Lesion conspicuity scores showed no significant differences among the three sequences for any reader (all *P*>0.017).

**Table 3:** Comparison of qualitative image quality assessments

	Reader 1				Reader 2				Reader 3				Interrater agreement
	MPRAGE	ZTE	ZTE-DL	*P*-value	MPRAGE	ZTE	ZTE-DL	*P*-value	MPRAGE	ZTE	ZTE-DL	*P*-value	
Noisiness	2.76 (0.930)	2.53 (0.547)	3.43 (0.628)	0.474^a^ 0.003^b^ <0.001^c^	3.05 (0.825)	2.60 (0.583)	3.68 (0.648)	0.019^a^ 0.009^b^ <0.001^c^	3.00 (0.795)	2.58 (0.494)	3.43 (0.543)	0.012^a^ 0.049^b^ < 0.001^c^	0.692^d^ 0.762^e^ 0.751^f^
Gray-white matter differentiation	3.21 (0.731)	2.80 (0.583)	3.53 (0.591)	0.008^a^ 0.278^b^ < 0.001^c^	3.16 (0.844)	2.58 (0.628)	3.18 (0.628)	0.001^a^ >0.99^b^ < 0.001^c^	3.32 (0.831)	2.68 (0.565)	3.45 (0.630)	0.001^a^ >0.99^b^ < 0.001^c^	0.563^d^ 0.603^e^ 0.668^f^
Artifacts	3.05 (1.025)	2.60 (0.735)	3.35 (0.614)	0.061^a^ >0.99^b^ < 0.001^c^	3.00 (0.946)	2.55 (0.669)	3.23 (0.724)	0.025^a^ >0.99^b^ < 0.001^c^	3.03 (0.986)	2.43 (0.628)	3.18 (0.543)	<0.001^a^ >0.99^b^ <0.001^c^	0.713^d^ 0.778^e^ 0.699^f^
Lesion conspicuity	3.25 (0.829)	3.14 (0.515)	3.57 (0.495)	>0.99^a^ 0.613^b^ 0.344^c^	3.33 (0.850)	2.86 (0.639)	3.43 (0.728)	0.205^a^ >0.99^b^ 0.289^c^	3.58 (1.115)	2.93 (0.703)	3.71 (0.795)	0.234^a^ >0.99^b^ 0.064^c^	0.622^d^ 0.603^e^ 0.771^f^
Overall image quality	3.08 (1.010)	2.60 (0.624)	3.43 (0.587)	0.016^a^ 0.815^b^ <0.001^c^	3.05 (0.916)	2.50 (0.592)	3.25 (0.622)	0.003^a^ >0.99^b^ <0.001^c^	3.08 (0.900)	2.50 (0.548)	3.28 (0.591)	0.001^a^ >0.99^b^ <0.001^c^	0.821^d^ 0.790^e^ 0.776^f^

A 5-point Likert scale was used for noisiness, gray-white matter differentiation, and overall quality (1, unacceptable; 5, excellent); artifacts, including both truncation and motion artifacts (1, unreadable; 5, no visible artifact); and lesion conspicuity (1, not visible; 5, highly distinct). All values are presented as mean scores with standard deviations in parentheses. Alphabetical footnotes indicate *P*-values from post hoc analysis using the Mann–Whitney *U* test with Bonferroni correction (^a^MPRAGE vs. ZTE, ^b^MPRAGE vs. ZTE-DL, ^c^ZTE vs. ZTE-DL) and weighted Cohen’s kappa values (^d^reader 1 vs. reader 2, ^e^reader 1 vs. reader 3, ^f^reader 2 vs. reader 3). *MPRAGE* magnetization-prepared rapid gradient-echo, *ZTE* zero echo time, *ZTE-DL* zero echo time with deep learning-based reconstruction

The weighted Cohen’s kappa values indicated substantial agreement between readers overall, except for moderate agreement between readers 1 and 2 in gray-white matter differentiation (*κ*=0.563), and almost perfect agreement between the same readers in overall image quality (*κ*=0.821).

### Quantitative analysis

The mean and standard deviation of the coefficient of variation for white matter, gray matter, and CSF in each sequence are summarized in Table 4. For both white and gray matter, the coefficient of variation was lowest in ZTE-DL, followed by ZTE and MPRAGE, indicating that ZTE-DL had the highest signal homogeneity (*P*=0.011 for MPRAGE vs. ZTE in white matter; all other comparisons, *P*<0.001). In CSF, ZTE-DL and ZTE showed lower coefficients of variation and greater signal homogeneity than MPRAGE, with no significant difference between ZTE and ZTE-DL (*P*=0.721 for ZTE vs. ZTE-DL; all other comparisons, *P*<0.001).

**Table 4:** Comparison of quantitative signal uniformity

	MPRAGE	ZTE	ZTE-DL	*P*-value
White matter	0.048 (0.014)	0.040 (0.005)	0.032 (0.006)	0.011^a^ <0.001^b^ <0.001^c^
Gray matter	0.038 (0.009)	0.031 (0.004)	0.024 (0.004)	<0.001^a^ <0.001^b^ <0.001^c^
Cerebrospinal fluid	0.088 (0.028)	0.050 (0.007)	0.052 (0.008)	<0.001^a^ <0.001^b^ 0.721^c^

Signal uniformity was assessed using the coefficient of variation, calculated as the standard deviation divided by the mean signal intensity. All values are presented as the mean coefficient of variation with standard deviations in parentheses. Alphabetical footnotes indicate *P*-values from post hoc analysis using the Mann–Whitney *U* test with Bonferroni correction (^a^MPRAGE vs. ZTE, ^b^MPRAGE vs. ZTE-DL, ^c^ZTE vs. ZTE-DL). *MPRAGE* magnetization-prepared rapid gradient-echo, *ZTE* zero echo time, *ZTE-DL* zero echo time with deep learning-based reconstruction

## Discussion

In this study, DL-based MRI reconstruction improved the image quality of ZTE acquired with the feed-and-wrap technique. Unsedated ZTE demonstrated lower overall image quality than sedated MPRAGE, whereas ZTE-DL achieved comparable quality to MPRAGE.

Reducing acoustic noise is a key strategy for minimizing motion during infant brain MRI, thereby reducing discomfort and image artifacts. Although secondary approaches such as earplugs or noise attenuators can be used, they have inherent limitations. Fundamental efforts focus on decreasing mechanical noise generated by gradient coil vibrations. The ZTE sequence is a crucial component of silent MRI; however, the delay between signal excitation and reception—caused by radiofrequency pulse duration, transmit–receive switching, and filter group delay—limits early data acquisition, resulting in a central k-space gap with missing data [11]. The central k-space gap and reduced peripheral resolution may diminish image contrast and sharpness, thereby compromising overall image quality. Additionally, the low flip angle reduces transverse magnetization, resulting in decreased signal and a lower SNR.

Several studies have investigated the utility of silent brain MRI in pediatric populations [6, 8]. These studies primarily focused on assessing myelination and quantifying gray-white matter contrast but did not provide a comprehensive evaluation of image quality. Compared to conventional sequences, the ZTE sequence is more prone to phase accumulation during readout caused by off-resonance effects such as main magnetic field inhomogeneity, tissue susceptibility differences, and fat–water chemical shift [10]. As a result, the types of image artifacts differ from those in conventional sequences, requiring careful assessment by radiologists. Additionally, due to the use of radial sampling, the sampling density is less uniform than in Cartesian sampling, and repeated averaging of central k-space data may cause localized blurring and streaking artifacts. These factors may explain the higher prevalence of artifacts observed in the ZTE sequence compared to MPRAGE in this study. While the ZTE sequence offers advantages in acoustic noise reduction, validation of its clinical utility is needed due to its inherent limitations in image quality.

However, direct comparison of image quality scores among the MPRAGE, ZTE, and ZTE-DL groups should be interpreted with caution. Sedation was administered prior to MPRAGE imaging, whereas the feed-and-wrap technique was used for ZTE imaging, making differences in sedation status and acquisition periods potential confounders. A previous study demonstrated that the feed-and-wrap technique, when applied to Cartesian sequences, yields acceptable but slightly degraded image quality due to motion, compared to that obtained with sedation [27]. Therefore, the superior image quality of MPRAGE compared to ZTE may be attributable to reduced motion resulting from sedation. The purpose of this study was not to compare imaging sequences under strictly controlled conditions, but rather to evaluate the clinical feasibility of DL-based reconstruction applied to the ZTE sequence. Our results suggest that ZTE-DL, when combined with the feed-and-wrap technique, provides a clinically viable alternative to the conventional approach of MPRAGE with sedation.

These findings were enabled by recent advances in DL-based MRI reconstruction, which learn complex relationships from undersampled k-space data and reconstruct high-quality images rapidly and accurately [20, 28, 29]. Previous studies have shown that DL-based reconstruction in 3-D T1-weighted pediatric brain MRI reduces acquisition time, improves image quality, and decreases artifacts [25], with similar benefits observed in 2-D T2-weighted imaging [16, 20]. However, those studies applied DL to Cartesian k-space data, either after undersampling [25] or by altering the reconstruction approach [16, 20]. Our findings demonstrate improved image quality and signal uniformity in T1-weighted images reconstructed from radially acquired k-space data using DL, supporting the effectiveness of combining DL with the ZTE sequence.

This study has several limitations. First, the MPRAGE and ZTE/ZTE-DL groups were retrospectively acquired as non-paired cohorts, introducing differences in patient characteristics and limiting the validity of direct image quality comparisons. To minimize such bias, a similarly sized cohort was selected based on a power calculation within the same NICU. Additionally, image quality was independently assessed by three radiologists with varying levels of experience to enhance statistical power. Second, because lesion conspicuity largely depends on lesion characteristics, it is challenging to determine whether differences in grading truly reflect differences in conspicuity. Major structural anomalies are highly conspicuous regardless of the sequence, while smaller lesions such as punctate white matter lesions or focal hemorrhages may be more affected by sequence differences. Moreover, since the readers were informed of the lesion locations, lesion conspicuity did not measure detection performance. Third, the DL-based MRI reconstruction algorithm was optimized for k-space data from a specific vendor, which limits its applicability across different vendors. Using DL in the image domain could be a possible solution to this limitation. Fourth, motion was not quantitatively assessed, and while sedation may influence examination failure or repetition rates, this was not reflected in the image quality scores. Fifth, the image quality of the ZTE sequence might also vary depending on the algorithm used to fill the central k-space gap, which was not examined in this study.

## Conclusion

The image quality of infant brain MRI acquired with the ZTE sequence combined with DL reconstruction was significantly improved compared to ZTE without DL reconstruction and was comparable to that of MPRAGE. Applying DL reconstruction to silent MRI with the ZTE sequence could be an effective approach to reduce the need for sedation in infants undergoing brain MRI.

## Data Availability

Data not included in the manuscript are available from the corresponding author upon reasonable request.
